# Preventing Black Ice Caused by Freezing Rain and Frost on Sensorless Rural Highways: Winter Maintenance in South Korea

**DOI:** 10.3390/s26134225

**Published:** 2026-07-03

**Authors:** Jinhwan Jang

**Affiliations:** Korea Institute of Civil Engineering and Building Technology, Goyang 10223, Republic of Korea; jhjang@kict.re.kr

**Keywords:** black ice, winter road maintenance, machine learning, XGBoost

## Abstract

Black ice poses a significant threat to drivers during winter due to its low visibility. Winter road maintenance personnel face continuous challenges in preventing its formation because of its high unpredictability. To address this issue, this paper proposes a practical strategy for effective winter road maintenance aimed at preventing black ice caused by freezing rain and frost. The strategy comprises three phases: black ice prediction, stakeholder notification, and anti-icing chemical application. The core of the strategy involves predicting black ice on rural highways that lack localized road weather sensors. Specifically, the prediction model relies exclusively on atmospheric data. The Extreme Gradient Boosting (XGBoost) algorithm was employed for prediction, achieving precision, recall, and F1 scores of 0.99 and outperforming Random Forest and Deep Neural Network models, which achieved F1 scores of 0.96 and 0.97, respectively. The XGBoost model’s hyperparameters were optimized using the DEPSO algorithm, improving its F1 score by approximately 0.03. Furthermore, a feature importance analysis was conducted to determine the relative contribution of various meteorological variables to black ice formation. To effectively disseminate predictive alerts to maintenance personnel and drivers, a smartphone-based system was developed. Finally, optimal spread rates for anti-icing chemicals, calibrated to pavement temperatures, are presented. The methodology proposed in this study can significantly enhance the efficiency of winter road maintenance on rural highways where localized road weather data are unavailable.

## 1. Introduction

Winter weather conditions significantly impact highway safety, with black ice representing one of the most perilous hazards for motorists. Characterized by a thin, transparent layer of ice, it drastically reduces tire traction while remaining virtually invisible to drivers. Consequently, more than 24 percent of weather-related vehicle crashes occur on snowy or icy pavements, resulting in over 1300 deaths and 116,800 injuries annually in the US [1]. In Korea, 3944 accidents occurred over the last five years, causing 95 deaths and 6589 injuries [2]. According to Korean statistics, the fatality rate under icy conditions is 1.8 times higher than under all other road conditions [3]. Winter road maintenance personnel face persistent challenges in anticipating and mitigating its formation. The highly unpredictable nature of black ice often renders traditional, reactive maintenance approaches insufficient, highlighting an urgent need for proactive prevention strategies. In particular, compared with snow-covered conditions, which are easily discernible to the naked eye, black ice caused by freezing rain and frost poses a greater threat to drivers because it is difficult to detect.

The difficulty of combating black ice is particularly pronounced on rural highways. Unlike major expressways or urban road networks that may be equipped with dense arrays of localized road weather sensors, rural routes typically lack such costly infrastructure. Without real-time, localized road surface data, predicting black ice formation becomes a formidable task, leaving these roadways exceptionally vulnerable to winter traffic accidents. Therefore, there is a critical need for predictive methodologies that can accurately forecast road conditions without relying on extensive localized sensor networks.

Recent advancements in machine learning offer promising avenues for addressing this data gap. By leveraging broadly available atmospheric data provided by meteorological agencies, it is possible to model and forecast localized road conditions. Algorithms such as Extreme Gradient Boosting (XGBoost) have demonstrated exceptional capability in handling complex, non-linear meteorological variables. When coupled with advanced optimization techniques—such as the Differential Evolution Particle Swarm Optimization (DEPSO) algorithm—these models can achieve high predictive accuracy and robustness, while also identifying the crucial meteorological factors that trigger black ice.

To address these challenges, this paper proposes a comprehensive and practical strategy for effective winter road maintenance, specifically targeting the prevention of black ice on rural highways. The core contribution of this study is a predictive methodology that relies exclusively on atmospheric data, bypassing the need for expensive road weather sensors. To achieve this, the paper outlines a cohesive operational procedure, detailing the following key phases:

- Black Ice Prediction: Utilizing a DEPSO-optimized XGBoost algorithm to predict black ice formation, accompanied by a feature importance analysis to determine the relative contribution of various meteorological variables.
- Stakeholder Notification: The development of a smartphone-based system to efficiently disseminate predictive alerts to both maintenance personnel and drivers.
- Anti-Icing Chemical Application: Providing guidelines for optimal spread rates calibrated to specific pavement temperatures through real-world experiments.

Ultimately, the methodology proposed in this study aims to significantly enhance the efficiency and safety of winter road maintenance operations in resource-constrained rural environments where localized road weather data are unavailable.

## 2. Literature Review

Developed nations worldwide are establishing multilateral strategies that combine Intelligent Transportation Systems (ITS), road engineering improvements, and institutional frameworks to address black ice—a critical threat to winter road safety. Black ice-responsive winter road maintenance cases from the US, Canada, Europe, and Japan are as follows.

The United States has standardized anti-icing techniques, which involve applying liquid chemicals before road freezing occurs. Particularly in high-snowfall regions like Wyoming, the Black Ice Detection and Warning Decision Support System (BIDWDSS) analyzes real-time weather and pavement data to inform drivers via Variable Message Signs (VMS) [4]. Canada operates a nationwide Road Weather Information System (RWIS) to precisely monitor pavement temperature and humidity. Concentrated surveillance is also conducted on high-risk zones, such as bridges and tunnel entrances, where freezing is most frequent [5]. European nations excel in developing automated systems that function without human intervention. Germany and Austria utilize Fixed Automated Spray Technology (FAST) at vulnerable locations, where sensors trigger the immediate application of brine upon detecting freezing conditions [6]. Japan actively utilizes road heating systems to directly control pavement temperature. Electric heating cables or hot water pipes are buried under slopes and intersections in major cities like Hokkaido to fundamentally prevent ice formation [7].

In recent years, leading nations have shifted their winter road safety paradigms from reactive snow removal to proactive (predictive) strategy that integrates big data and sensor engineering. Technological approaches adopted by various countries for the detection and forecasting of black ice are as follows.

The United States has advanced its predictive capabilities through the Maintenance Decision Support System (MDSS), which integrates meteorological forecasting with thermodynamic physical models of road surfaces [8]. Given its extreme low-temperature environments, Canada excels in thermal mapping technology, which analyzes the thermal characteristics of individual road segments. This involves scanning road surfaces to identify cold spots that freeze faster than surrounding areas due to topographical or structural features [9]. European nations lead in non-invasive sensor technologies that assess road conditions without physical pavement disruption. This information is linked with AI-driven black ice prediction minute-by-minute climate changes at specific sites like bridges or tunnel entrances [10,11,12,13,14]. Japan actively utilizes high-resolution CCTV image recognition and big data from vehicle maneuvers. AI algorithms detect black ice formation [15].

However, previous studies have primarily developed black ice prediction and response methods for highways equipped with road weather sensors. To date, there have been no documented cases in which black ice was predicted using only atmospheric data on rural roads without road weather sensors, nor have response methodologies been developed based on such predictions. Nevertheless, wintertime traffic crashes caused by black ice occur frequently on rural roads, highlighting the need for further research in this area. Therefore, this study aims to develop black ice prediction and response methods that can be applied to rural highways where road weather sensors are not installed.

The novelty of this study lies not only in its predictive methodology but also in the development of a comprehensive framework for utilizing black ice prediction information from the perspectives of both drivers and road administrators. Furthermore, by recommending appropriate deicing agent application rates based on road weather conditions, this study addresses both winter road safety and environmental sustainability.

## 3. Predicting Black Ice-Prone Segments Using Weather Forecast Data

### 3.1. Data

**Figure 1:**
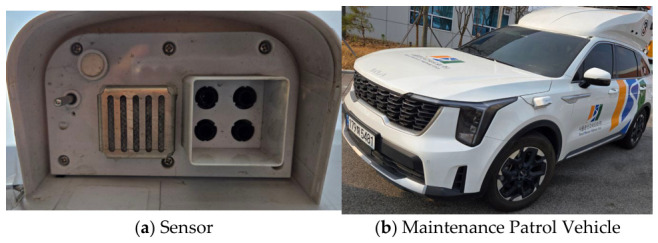
Pavement Temperature Sensor Installed on Maintenance Patrol Vehicle.

This study utilized a dataset comprising atmospheric variables—air temperature, relative humidity, precipitation, and wind speed—alongside pavement temperatures collected via maintenance patrol vehicles. The atmospheric data served to predict black ice-prone segments, while pavement and dew point temperatures were combined to establish baseline data. Pavement temperature data were aggregated into standard links defined by intersections, bridges, and tunnels. The procedures for generating these baselines, as well as the specifications of the applied pavement temperature sensors, are described elsewhere [16,17]. Pavement surface temperature was measured using a non-contact infrared sensor (refer to Figure 1), which collected data at 0.2-s intervals. Its narrow 5° field of view enabled highly accurate measurements of road surface temperature. At 0 °C, the measurement uncertainty was ±0.3 °C, as verified by a Korean accredited testing laboratory. Data collection spanned from December 2024 to February 2025 across an approximately 12,000 km stretch of national highways on the Korean Peninsula. These routes predominantly feature four lanes and an 80 km/h speed limit. The finalized dataset contains 159,424 records. Each record integrates pavement temperature, air temperature, relative humidity, precipitation, wind speed, and a derived dew point temperature calculated via the Magnus formula. All data were collected during nighttime hours (23:00 to 07:00), coinciding with active maintenance patrols targeting black ice mitigation. The pavement temperature data collected by patrol vehicles were transmitted to and stored on the winter road maintenance system server in real time. At each corresponding timestamp, atmospheric weather data were retrieved from the Korea Meteorological Administration server and stored in the same database table. All timestamps were synchronized using GPS time.

**Figure 2:**
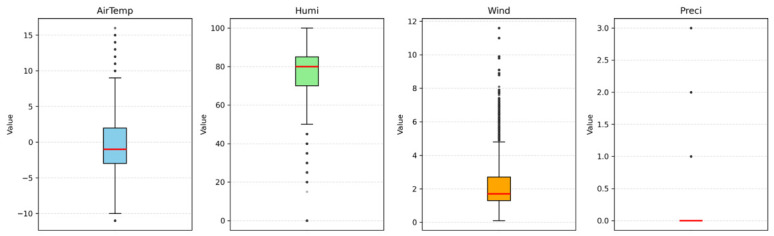
Boxplot of Atmospheric Data.

The statistical summary (see Table 1) of the collected dataset reflects typical winter weather conditions in South Korea. While pavement and air temperatures exhibited broadly similar trends, pavement temperatures were consistently lower than air temperatures. This discrepancy is likely attributable to spatial differences in data collection. Specifically, air temperature forecasts are provided at a 5 × 5 km grid resolution across the Korean Peninsula, whereas pavement temperatures were measured directly on the road surface via vehicle-mounted infrared sensors. Because approximately 70% of South Korean territory is mountainous and highways frequently traverse these higher elevations, road surface temperatures tend to be lower than atmospheric forecasts, which are often calibrated for residential areas on level terrain. Furthermore, all recorded precipitation during the study period consisted exclusively of rainfall. Owing to South Korea’s climatic characteristics, winter precipitation is relatively infrequent and generally ranges from 1 to 3 mm/hr per rainfall event. Data collection patrols were suspended during snowfall events, as vehicles were reassigned to active snow plowing and de-icing operations. Figure 2 shows box plots of the atmospheric weather data.

### 3.2. Black Ice Prediction Model

**Figure 3:**
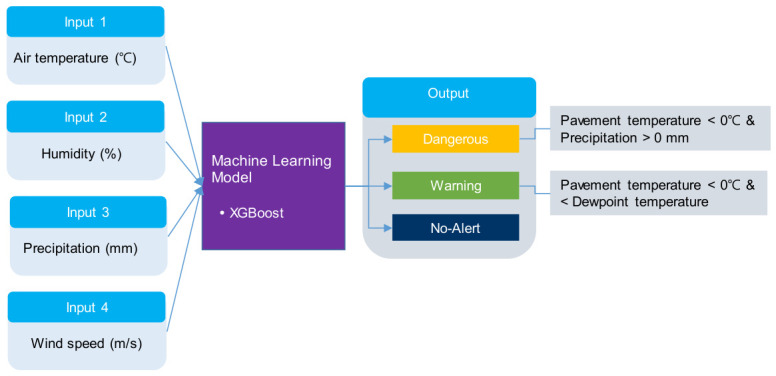
Building Blocks of Machine Learning Model for Black Ice-Prone Segments.

The XGBoost model was employed to predict black ice-prone segments. According to a previous study [18], XGBoost, a type of boosting model, outperforms other models such as Random Forest (a bagging model) and Deep Neural Networks. Figure 3 shows the block diagram of the developed model. The model utilized Softmax classification (dangerous, warning, and no-alert) rather than binary classification for efficient black ice mitigation activities.

In this study, cases in which the pavement temperature was below 0 °C and equal to or lower than the dew-point temperature were classified as “warning,” as such conditions may indicate the potential formation of frost or black ice through water vapor deposition or the freezing of condensed water. In addition, cases in which precipitation was observed while the pavement temperature was below 0 °C were classified as “dangerous,” because moisture supplied to the road surface may freeze or solid precipitation may accumulate. However, based on previous studies [19,20], these criteria were applied as a preventive risk classification scheme rather than as definitive indicators of road icing. It should be noted that the black ice prediction framework developed in this study is intended to support efficient preventive winter road maintenance.

All data were standardized, and the dataset was randomly split into training, validation and testing sets at a 0.7:0.15:0.15 ratio according to earlier relevant studies [21,22,23]. To mitigate bias toward the majority class caused by differences in sample size across classes, class weights inversely proportional to class frequencies were calculated using Equation (1) and incorporated into the XGBoost training loss function. The resulting weights for the three classes were 0.431 (123,368 samples), 2.100 (25,300 samples), and 4.941 (10,756 samples), respectively. These weights were applied only to the training set.

(1) $$w_{i}=\frac{N}{Kn_{i}}$$

where

- N: Total number of samples,
- K: Number of classes,
- $n_{i}:$ Number of samples in class i

To achieve optimal performance, the XGBoost model requires the optimization of its hyperparameters, including number of estimators, learning rate, and max depth of decision tree. To accomplish this, the Differential Evolution Particle Swarm Optimization (DEPSO) algorithm was employed. DEPSO, expressed in Equation (2), is a highly effective hybrid algorithm that combines the rapid convergence of Particle Swarm Optimization (PSO) with the strong global search capabilities of Differential Evolution (DE). It is widely utilized in machine learning for hyperparameter tuning because it resists getting trapped in local optima [24]. During DEPSO optimization, macro-averaged F1 score and balanced accuracy, which assign equal importance to each class, were used in addition to overall accuracy. No resampling was applied to the validation or test datasets. For DEPSO optimization, the hyperparameter search ranges were set to a maximum of 100 estimators, 0.01–0.20 for the learning rate, and 3–10 for the maximum tree depth. To mitigate overfitting, early stopping rounds was set to 10.

(2) $$X_{i}\left(t+1\right)=X_{i}\left(t\right)+\omega V_{i}\left(t\right)+c_{1}r_{1}\left({\arg min}_{Y\in \left\{P_{k}\left(t\right),U_{i}\left(t\right)\right\}}f\left(Y\right)-X_{i}(t)\right)+c_{2}r_{2}(P_{g}\left(t\right)-X_{i}\left(t\right))$$

where

- $X_{i}\left(t+1\right)\;\mathrm{and}\;X_{i}(t):$ The set of hyperparameters being optimized at iteration t+1 and t, respectively,
- $V_{i}\left(t\right):$ The current velocity vector of particle I,
- ω: The inertia weight,
- $c_{1}:$ The cognitive learning factor scaling the particle’s tendency to return to its own historical best-performing configuration,
- $c_{2}:$ The social learning factor scaling the particle’s tendency to move toward the best-performing configuration found by the entire swarm,
- $r_{1},r_{2}:$ Random vectors uniformly distributed in the range [0, 1],
- f: The objective function for minimization,
- $P_{i}(t):$ The traditional personal best position discovered by particle I up to iteration t,
- $U_{i}(t):$ The trial vector generated through the Differential Evolution process, and
- $P_{g}\left(t\right):$ The global best position.

**Figure 4:**
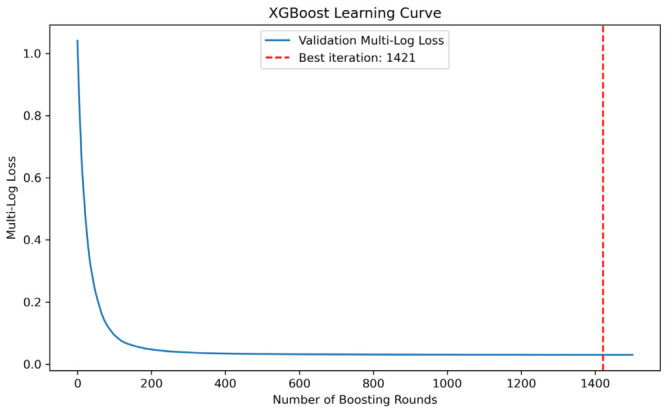
Learning Curve of XGBoost.

Consequently, the optimized hyperparameters for the XGBoost model were determined as follows. Figure 4 illustrates the learning curve of the trained model. The multi-class log loss (mlogloss) metric was utilized as the loss function to correspond with the Softmax classification approach.

- Number of estimators (number of boosting rounds): 34
- Learning rate: 0.0562
- Max depth of decision tree: 8

The developed model was evaluated using the metrics defined in Equations (3)–(6). To account for the multi-class nature of the Softmax classification, macro-averaged metrics were utilized for precision and recall. As shown in Table 2 and Table 3, the model achieved precision, recall, and F1 scores of 0.99, demonstrating excellent predictive performance. DEPSO optimization improved the F1 score by approximately 0.03 (Table 4). Furthermore, as shown in Table 5, XGBoost outperformed the Random Forest and Deep Neural Network models by 0.01–0.03 in terms of F1 score.

(3) $$Accuracy=\frac{TP+TN}{TP+TN+FP+FN}$$

(4) $$Precision=\frac{TP}{TP+FP}$$

(5) $$Recall=\frac{TP}{TP+FN}$$

(6) $$Macro\;Precision\;(Recall)=\frac{{Precision\;(Recall)}_{A}+{Precision\;(Recall)}_{B}+{Precision(Recall)}_{C}}{3}$$

where:

- TP is the number of true positives,
- TN is the number of true negatives,
- FP is the number of false positives, and
- FN is the number of false negatives.

When road icing risk levels are classified using air temperature, humidity, wind speed, and precipitation, threshold effects and interactions among variables become important. Because these variables constitute structured tabular data, XGBoost is likely to outperform DNNs and may form more precise decision boundaries than Random Forest. In particular, precipitation data may exhibit strong skewness and heavy-tailed distributions, as most observations are zero while a small number contain relatively large values. As such irregularities increase, XGBoost, a boosting-based ensemble model, has been reported to outperform bagging-based Random Forest and DNN models [25].

### 3.3. Factors Affecting Black Ice Formation

Understanding the atmospheric factors that affect black ice formation is crucial for road managers to mitigate potential hazards. To analyze the quantitative contributions of these atmospheric factors to black ice formation, Shapley values were computed. The Shapley value, expressed in Equation (7), originates from cooperative game theory. In machine learning, it serves as a method to fairly allocate and quantify the contribution of each feature to the model’s final prediction. The core concept involves calculating the difference in a prediction with and without a specific feature (the marginal contribution) across all possible feature combinations and then averaging these marginal contributions [26].

(7) $$\emptyset_{i}=\sum_{S\subseteq N\left\{i\right\}}\frac{\left|S\right|!(\left|N\right|-\left|S\right|-1)!}{\left|N\right|!}\left[v\left(S\cup \left\{i\right\}\right)-v(S)\right]$$

where

**Figure 5:**
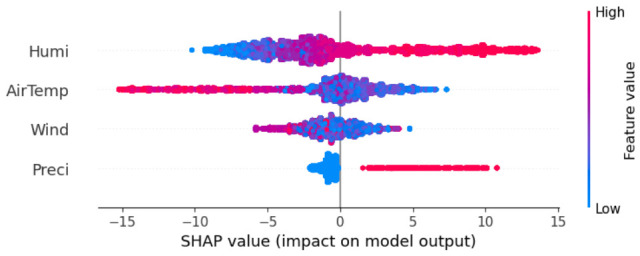
SHAP Value Visualization of Factors Affecting Black Ice Risk.

- $\emptyset_{i}:$ The final contribution (Shapley Value) of the specific feature,
- i:A single, specific feature (or variable),
- S: All possible subsets (combinations),
- N: The set of all features,
- $\left|N\right|,\left|S\right|:$ The number of features in the total set and the subset, respectively,
- $\frac{\left|S\right|!(\left|N\right|-\left|S\right|-1)!}{\left|N\right|!}:$ Weight, and
- $\left[v\left(S\cup \left\{i\right\}\right)-v(S)\right]:$ Marginal contribution,

Figure 5 presents the SHAP-based interpretation of the effects of the meteorological variables on black ice risk prediction. In Figure 5, the horizontal axis represents the SHAP value, which indicates the magnitude and direction of each variable’s contribution to the model output. Higher humidity values are predominantly associated with positive SHAP values, indicating that increasing humidity tends to increase the predicted risk of black ice formation. In contrast, higher air temperatures are generally associated with negative SHAP values, whereas lower temperatures contribute positively to black ice risk. High precipitation values also exhibit strongly positive SHAP values, demonstrating that precipitation substantially increases the predicted risk by supplying moisture to the pavement surface. Wind speed has a comparatively smaller influence; however, lower wind speeds tend to be associated with slightly positive SHAP values, suggesting a modest increase in predicted black ice risk under low-wind conditions.

**Figure 6:**
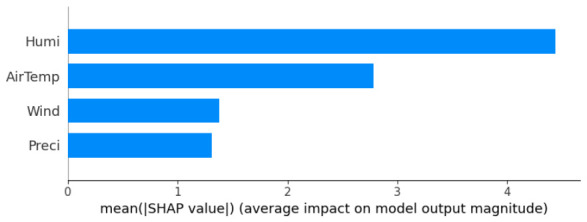
Feature importance based on SHAP values.

Figure 6 summarizes the global importance of the variables using their mean absolute SHAP values. The horizontal axis represents the mean absolute SHAP value, with larger values indicating a greater overall influence on model predictions, irrespective of whether the effect is positive or negative. The vertical axis lists the meteorological variables in descending order of importance. Humidity exhibits the greatest influence, followed by air temperature, wind speed, and precipitation. Although precipitation has a slightly lower mean absolute SHAP value than wind speed, Figure 5 shows that its effect becomes particularly strong when precipitation is observed.

**Figure 7:**
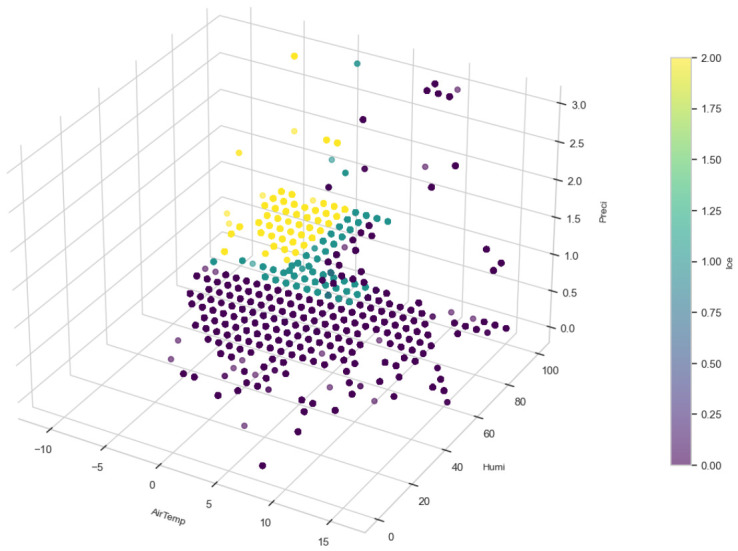
Black Ice Risk 4D Scatter Plot Based on Weather Data.

Figure 7 presents a four-dimensional scatter plot of black ice risk classifications based on air temperature, humidity, and precipitation. The three spatial axes represent air temperature, humidity, and precipitation, respectively, while point color represents the predicted risk class, providing the fourth dimension. Each point corresponds to an individual observation. Yellow points indicate hazardous conditions, green points indicate cautionary conditions, and purple points indicate no-alert conditions. Hazardous and cautionary cases are concentrated mainly under combinations of relatively low air temperature, high humidity, and measurable precipitation. Overall, Figure 5, Figure 6 and Figure 7 demonstrate that black ice risk is determined by the combined effects of multiple meteorological variables, particularly humidity, air temperature, and precipitation, rather than by any single variable alone.

## 4. Alerting Predicted Black Ice Segments

**Figure 8:**
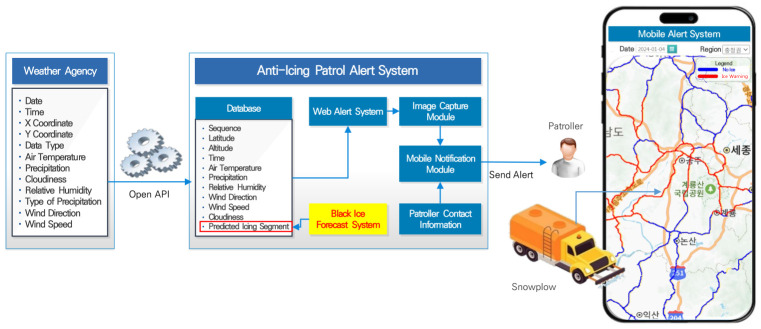
Alerting Black Ice Warning and Dangerous Sections to Maintenance Personnel through Multimedia Messaging Service.

The predicted black ice information described above is disseminated to maintenance personnel via smartphones, enabling them to perform preventive maintenance against black ice formation. Figure 8 illustrates the architecture of the black ice alert system currently operating in South Korea. Atmospheric data relayed via an Application Programming Interface is used to generate black ice forecasts. These forecasts cover every rural highway that lacks road weather sensors. Once a black ice alert is generated for a warning or dangerous segment, preventive measures are taken, such as applying anti-icing chemicals or displaying warning messages on Variable Message Signs.

**Figure 9:**
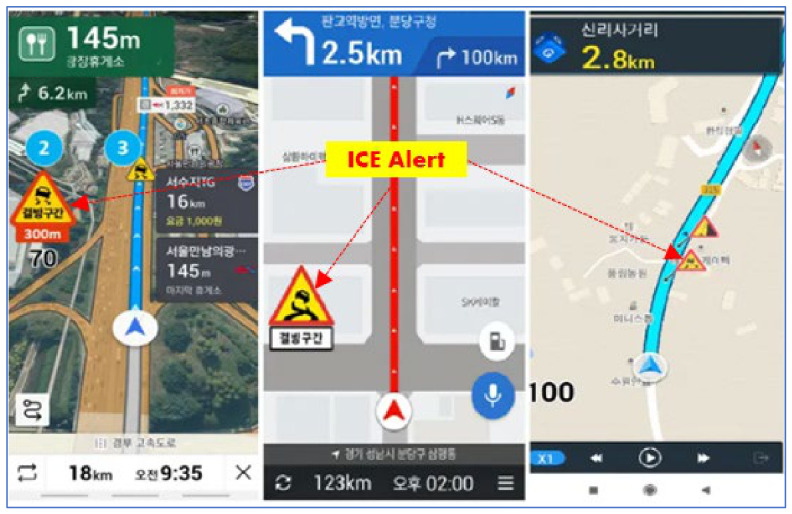
Alerting Dangerous Sections of Black Ice to Drivers through Car Navigation Systems.

The predicted information is also provided to drivers through three types of smartphone-based Car Navigation Systems (CNSs) (Figure 9). In South Korea, approximately 80% of drivers use smartphone-based CNSs. If a dangerous segment exists along a route, a warning message and corresponding icons are displayed on the CNS 300 m ahead of the affected area. After one season of operation (2025–2026), most drivers expressed satisfaction with the alert information provided.

## 5. Anti-Icing Chemical Application Strategy

**Figure 10:**
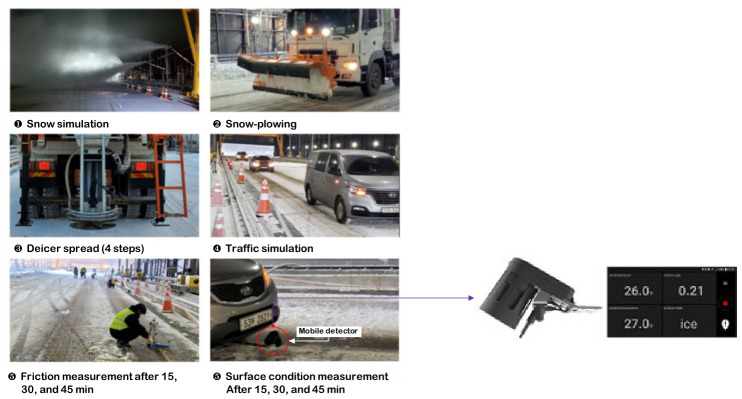
Experiment to Determine the Optimal Spread Rate.

Based on the forecast, applying an optimal amount of anti-icing chemicals is essential not only to prevent black ice on segments with warning or dangerous alerts, but also to protect the environment. To determine the appropriate application rate, the pavement temperature is first measured using an infrared sensor mounted on a snowplow or patrol vehicle. Liquid anti-icing chemicals (a 23% brine solution) are then applied at the rates specified in Table 6, depending on current weather conditions (e.g., fog, frost, or rain). These application rates were derived from field experiments (Figure 10); comprehensive details regarding the experimental methodology and derivation processes are available in a separate publication [27].

- Measure pavement temperature using pavement temperature sensor attached to snowplow.
- Frost or freezing fog: Apply anti-icing chemical on the roadway sections with pavement temperature lower than zero degree Celsius and dew point temperature.
- Freezing rain: Apply anti-icing chemical on the roadway sections with pavement temperature lower than zero degree Celsius while raining.

## 6. Conclusions and Future Studies

Black ice presents a severe and unpredictable hazard for winter motorists, with the challenge being particularly acute on rural highways that typically lack dense networks of localized road weather sensors. To address this critical vulnerability, this study proposed a proactive and comprehensive winter road maintenance strategy that bypasses the need for costly localized infrastructure. By relying exclusively on widely available atmospheric data, the proposed methodology offers a practical approach to mitigating black ice risks in resource-constrained environments.

At the core of this strategy is a predictive model utilizing the Extreme Gradient Boosting (XGBoost) machine learning algorithm, which was further optimized using the Differential Evolution Particle Swarm Optimization (DEPSO) algorithm to achieve a robust prediction accuracy of 99%. Through a feature importance analysis employing Shapley values, the study quantified the impact of various atmospheric variables on road conditions. The findings demonstrated that humidity and air temperature are the primary factors driving black ice formation, while precipitation and wind speed also significantly influence the risk levels.

To ensure the predictive data translates into actionable safety measures, a smartphone-based notification system was developed to deliver real-time alerts to maintenance personnel and to drivers via CNSs. Furthermore, the study provides specific, calibrated guidelines for the application of anti-icing chemicals based on pavement temperatures and weather conditions derived from field experiments. Ultimately, the integration of atmospheric data-driven predictions, automated stakeholder alerts, and optimized chemical application strategies can significantly enhance the safety and efficiency of winter maintenance operations on sensorless rural highways.

However, the black ice forecasts were evaluated using pavement and dew point temperature data—black ice is assumed to form when the pavement temperature is lower than the dew point and below zero degrees Celsius. Although the baseline data are grounded in stringent physical theory [28,29], they were not confirmed by real-world measurements. Therefore, a follow-up study will be conducted during the 2026–2027 winter season to evaluate field-measured reference data collected using high-cost, sensitive optical pavement sensors.

## Figures and Tables

**Table 1:** Statistics of Collected Data.

Statistics	Pavement Temperature (°C)	Air Temperature (°C)	Relative Humidity (%)	Precipitation (mm)	Wind Speed (m/s)
Mean	−3.5	−2.7	74	1.0	2.2
Std. dev.	4.0	3.8	15	0.1	1.4
Min.	−19.6	−18.0	15	1.0	0.1
25%	−6.0	−5.0	65	1.0	1.2
50%	−3.7	−3.0	75	1.0	1.7
75%	−1.0	0	85	1.0	2.7
Max.	14.0	14.0	100	3.0	10.7

**Table 2:** Classification Result.

	Precision	Recall	F1-Score
0 (No-alert)	0.9968	0.9899	0.9934
1 (Warning)	0.9526	0.9845	0.9683
2 (Dangerous)	1.0000	1.0000	1.0000
Accuracy			0.9898
Macro Avg.	0.9831	0.9915	0.9872
Weighted Avg.	0.9900	0.9898	0.9898

**Table 3:** Confusion Matrix.

		Prediction		
		0 (No-Alert)	1 (Warning)	2 (Dangerous)
Real	0 (No-alert)	18,320	186	0
	1 (Warning)	59	3736	0
	2 (Dangerous)	0	0	1631

**Table 4:** Performance of XGBoost with and without DEPSO Optimization.

	XGBoost with DEPSO Optimization	XGBoost Without DEPSO Optimization
F1-score	0.9898	0.9632

**Table 5:** Performance Comparison across Models.

	XGBoost	Random Forest	DNN
F1-score	0.9898	0.9614	0.9736

**Table 6:** Recommended Application Rate.

Cases		Pavement Temperature (°C)	Application Rate (g/m^2^)
Anti-icing	Before Snow	≤2 °C	14.3
	Before Rain (Freezing Rain)	≤2 °C	17.7 (44.4)
	Frost or Fog	≤0 °C	11.1
Snowing		≥−2 °C	13.6
		−2 °C to −10 °C	27.2
		≤−10 °C	40.8
Packed Ice		≥−5 °C	35.7
		<−5 °C	42.9

## Data Availability

The data presented in this study are available on request from the corresponding author due to proprietary restrictions.
